# Entropy-Driven Volatility Prediction for ETF Quantitative Investment in the Chinese Stock Market: A Machine Learning Framework for Barbell Strategy Optimization

**DOI:** 10.3390/e28091035

**Published:** 2026-09-20

**Authors:** Keyue Yan, Zihuan Yue, Qiqiao He, Ying Li

**Affiliations:** 1 School of Data Science and Artificial Intelligence, Guangdong University of Finance, Guangzhou 510521, China; 2 School of International and Continuing Education, Beijing Institute of Technology (Zhuhai), Zhuhai 519088, China; 3 School of Computer Science and Artificial Intelligence, Foshan University, Foshan 528225, China

**Keywords:** volatility prediction, time series, quantitative trading, machine learning

## Abstract

Volatility is a core determinant of risk management and return optimization in financial investment. We develop an integrated stock-volatility prediction framework that couples multi-dimensional entropy indicators with machine learning models and links the resulting forecasts to a dynamic Barbell Strategy. The strategy controls drawdowns while retaining upside and remains feasible for individual investors. Using data for the Chinese CSI 300, CSI 500, and CSI 1000 index ETFs and a government bond ETF, we construct predictive features and estimate Yang–Zhang Volatility. The framework incorporates four entropy indicators—Shannon Entropy, Fuzzy Entropy, Permutation Entropy, and Dispersion Entropy—and evaluates model performance under 10-day, 15-day, and 20-day prediction and rebalancing frequencies. The empirical results reveal that the volatility forecasting model for the CSI 1000 has the highest R Squared. In practical trading applications, the Random Forest achieves the optimal risk-adjusted returns, and the 15-day and 20-day portfolio frequencies realize a better trade-off between return and risk control.

## 1. Introduction

In the increasingly complex global financial market, the Exchange-Traded Fund (ETF) has become among the fastest-growing investment instruments in increasingly complex global markets. Their appeal lies in broad asset coverage, low transaction costs, flexible trading mechanisms, and high transparency. By tracking underlying assets across equities, bonds, and commodities, ETFs serve both individual investors seeking risk diversification and institutions building large allocation portfolios [1,2]. ETF volatility is a complex phenomenon driven by interdependent factors. First, it is substantially affected by systemic market drivers, such as global economic cycles, cross-border monetary policies implemented by central banks, and international capital flows. Second, ETF volatility exhibits prominent time-varying characteristics and volatility clustering behaviors [3]. More importantly, the structural properties and trading mechanisms of ETFs can amplify volatility: low costs and high liquidity attract short-term speculation, and arbitrage linkages between ETFs and their constituents propagate trading shocks across markets, intensifying volatility spillovers [4]. These intricate dynamics pose challenges to ETF investment and risk management, making accurate volatility forecasting essential for risk pricing, strategy formulation, and return optimization. A further challenge in quantitative trading and portfolio investment lies in dynamically adjusting asset allocation based on real-time volatility signals, controlling downside risks while capturing potential excess returns.

Volatility forecasting is a long-standing core challenge in quantitative finance, historically dominated by statistical models. Early analytical approaches like Autoregressive (AR), Moving Average (MA), Autoregressive Integrated Moving Average (ARIMA), and the Generalized Autoregressive Conditional Heteroskedasticity (GARCH) models established a foundation for volatility forecasting by capturing volatility clustering [5,6]. However, their rigid parametric assumptions limit their ability to characterize the nonlinear dynamics of financial markets, a drawback especially severe for multi-asset portfolios such as ETFs [7]. In recent years, the proliferation of big data techniques and machine learning has shifted the research paradigm toward data-driven methods. Machine learning models are capable of extracting latent complex patterns and cross-factor interaction effects from multidimensional financial data, such as historical prices and trading volumes. Existing studies have verified that machine learning-based models, including Neural Networks (NNs), can achieve superior forecasting performance relative to traditional linear models, yielding significant improvements in volatility prediction accuracy [8]. Advanced deep learning models, such as Gated Recurrent Units (GRUs), Long Short-Term Memory (LSTM), and Temporal Convolutional Networks (TCNs), exhibit distinct advantages in modeling long-range temporal dependencies [9,10]. Despite these advancements, machine learning and deep learning approaches suffer from a prevalent limitation: they focus on fitting historical data patterns primarily, while neglecting the intrinsic statistical properties of financial markets. As high-entropy systems, financial markets exhibit dynamically evolving, stochastic information flows that complicate reliable volatility forecasting. Entropy, as a fundamental measure for quantifying systemic uncertainty, offers a promising tool to address this limitation [11,12]. Under Shannon information theory, higher entropy indicates greater disorder and lower predictability [13]. In financial markets, the entropy dynamics of asset volatility is a powerful tool for quantifying the evolution of market uncertainty. Entropy theory not only provides an information-based perspective for interpreting market dynamics, but also delivers practical advantages for forecasting model optimization. Specifically, entropy-based methods can be implemented in the feature engineering procedure of predictive modeling [14] or adopted as a core paradigm for constructing innovative volatility indicators [15]. These applications enable the comprehensive integration of multi-dimensional price and volume information and facilitate the identification of heterogeneous market states, thereby enhancing the overall predictive capability of forecasting models. Despite progress in entropy and volatility research, a quantitative framework dedicated to ETF volatility forecasting for multi-asset portfolios remains underdeveloped. The existing literature focuses largely on prediction tasks or on controlling overall investment volatility, with limited integration of forecasts into practical trading strategies. As a result, the practical value of volatility forecasts is underexploited in quantitative investment.

To fill these gaps, this research poses four research questions, and each research question is addressed by a dedicated set of experiments in Section 4:

1. Do multi-dimensional entropy indicators improve the accuracy of Yang–Zhang Volatility forecasting for Chinese index ETFs, and how does this improvement vary across the Large-Cap, Mid-Cap, and Small-Cap market segments?
2. Which combinations of machine learning models, entropy indicators, and prediction windows deliver the most accurate volatility forecasts?
3. Can entropy-driven volatility forecasts be effectively translated into a dynamic Barbell Strategy that improves risk-adjusted returns, and which rebalancing frequency provides the most favorable risk–return trade-off?
4. To what extent is forecasting accuracy aligned with trading performance in the proposed strategy?

To answer these questions, we propose an integrated ETF volatility forecasting framework combining multidimensional entropy indicators with machine learning. Based on the forecasts, we develop a dynamically adjusted Barbell Strategy that unifies multi-entropy quantification, multi-window volatility prediction, and dynamic strategy optimization. Our volatility-driven strategy differs from traditional volatility-based risk-control approaches in configuration logic. From the perspective of portfolio construction, traditional volatility-based risk control strategies aim to control overall portfolio volatility at a predefined target without imposing strict restrictions on asset structural allocation. In contrast, our approach is a structurally constrained and dynamically rebalanced method in the standard Barbell Strategy, where volatility solely serves as a timing signal for weight adjustment of assets at the two ends of the Barbell allocation structure. In terms of adjustment objectives, traditional volatility-based risk control strategies prioritize the temporal stability of portfolio volatility. Such strategies may even employ leverage to amplify returns alongside underlying risks when market volatility falls below the preset threshold. Unlike these mechanisms, our framework does not constrain portfolio volatility at a fixed level. Instead, it leverages volatility signals to dynamically align portfolio risk with prevailing market conditions. This design retains the inherent tail-risk hedging advantages of the Barbell structure while effectively improving the risk-adjusted returns.

The research targets one-day-ahead volatility forecasts over three horizons: 10-day, 15-day, and 20-day, which also serve as rebalancing frequencies for the dynamic Barbell strategy. These settings are grounded in the institutional features of the Chinese market, which comprises five trading days per week. Specifically, the 10-day, 15-day, and 20-day windows correspond to biweekly, three-week, and approximately monthly trading cycles, respectively. The window design captures both short-term and medium-term volatility dynamics comprehensively, establising a hierarchical experimental framework that supports the cross-cycle prediction evaluation and comparative analysis of alternative portfolio rebalancing frequencies. We further introduce four entropy metrics, Shannon Entropy, Fuzzy Entropy, Permutation Entropy, and Dispersion Entropy, as predictive features, combined with historical prices, volumes, and technical indicators. Comparative evaluation across settings identifies optimal model–feature combinations and clarifies the associations among entropy indicators, cross-cycle volatility, and model performance, providing quantitative evidence for trading strategy optimization. In summary, the main contributions of this study are as follows:

1. We develop an integrated entropy-driven framework for Yang–Zhang Volatility forecasting that couples four entropy indicators with machine learning models and systematically evaluates their performance across market-capitalization segments and prediction windows.
2. We bridge volatility forecasting and portfolio management by translating multi-window volatility forecasts into a Barbell Strategy, in which volatility serves as a timing signal for structural weight adjustment, which improves risk-adjusted returns.
3. Based on a walk-forward experiment spanning 2015–2025 that covers multiple market regimes, we provide comprehensive empirical evidence on how entropy features, model choice, and rebalancing frequency jointly affect forecasting and trading performance, and reveal a non-trivial divergence between forecasting accuracy and trading efficacy.

Our empirical results demonstrate that entropy features consistently improve volatility forecasting across all market-capitalization segments. Forecasting accuracy generally improves as the prediction window extends from 10 to 20 days, with Shannon Entropy performing best at the 20-day window. In trading applications, the Random Forest model achieves the highest risk-adjusted returns. Moreover, the 15-day and 20-day rebalancing frequencies realize a better trade-off between return and risk control than the 10-day frequency.

The remainder of this research is organized as follows. Section 2 reviews the literature on financial volatility forecasting, machine learning in finance, and entropy-based methods. Section 3 details the methodology, including data preprocessing, entropy-driven feature engineering, forecasting model configuration, and the design of the volatility-driven trading strategy. Section 4 presents empirical results for both benchmark and forecasting models and evaluates the conventional and composite strategies. Section 5 concludes with core findings and future directions.

## 2. Related Works

This section reviews machine learning applications in stock time series analysis, tracing their evolution from price movement prediction to asset volatility forecasting, with emphasis on volatility modeling and entropy-based methods. And then we discuss the research progress and persistent limitations in portfolio investment applications and identify the research gaps this work addresses.

### 2.1. Machine Learning in Financial Time Series Forecasting

Machine learning enables systems to learn latent patterns through algorithmic iteration, supporting autonomous prediction and data-driven decision-making. Forecasting asset price dynamics has been a central focus, with studies extensively validating and optimizing models for stock-price prediction [16,17,18] and improving performance with external information such as news sentiment and knowledge-graph features [19]. Despite this progress, financial time series forecasting faces substantial challenges from market stochasticity and noise. Short-term price movements are jointly driven by macroeconomic conditions, policy adjustments, sentiment shifts, and cross-market capital flows. Within short forecasting horizons, machine learning models are highly susceptible to overfitting trivial market noise as stable predictive patterns, which substantially undermines their out-of-sample generalization ability [20,21]. Although existing studies have attempted to enhance price prediction accuracy through feature engineering and advanced machine learning architectures, considerable limitations persist in forecasting highly volatile financial assets. For example, Shui et al. combined feature engineering with LightGBM to predict volume-weighted average prices, but volatile assets remain challenging for machine learning [22]. In a comprehensive review, Wu evaluated the performance of LSTM, CNN, and Transformer models in cryptocurrency price prediction and documented non-negligible prediction errors across all mainstream architectures [23].

To address these limits, scholars have shifted from direct price prediction to volatility forecasting, using machine learning to capture the magnitude and temporal evolution of asset-price fluctuations [24]. Volatility series are more regular and tractable than asset returns and short-term price variations. A well-documented stylized fact in finance is volatility clustering, whereby high-volatility periods tend to be followed by subsequent high volatility, and low-volatility periods persistently coincide with low market fluctuation. This phenomenon endows volatility series with significant long-term memory, a distinctive property that is largely absent from price return time series [25]. This stable dependence makes volatility a more suitable target for machine learning to identify and generalize inherent market patterns. In cryptocurrency markets, Brauneis and Saliner compared the Heterogeneous Autoregressive (HAR) model with several machine learning approaches, confirming that machine learning models generally outperformed the HAR benchmark and can capture the nonlinear link between investor sentiment and market volatility [26]. In equity markets, Kumar et al. built a hybrid framework combining optimized Variational Mode Decomposition (VMD) with deep learning, achieving superior realized volatility prediction for major indices [27]. Furthermore, Yan and Li unified time series analysis and machine learning for volatility forecasting and embedded the model in quantitative trading strategies [28]. Collectively, this literature confirms that machine learning models, especially hybrid architectures integrating econometric and signal-processing methods, exhibit unique advantages in characterizing the complex nonlinear dynamics of financial time series. Such hybrid frameworks can effectively improve forecasting accuracy, thereby providing a robust and reliable analytical foundation for financial risk management and quantitative investment.

### 2.2. Volatility Estimation and Forecasting

Typical volatility metrics include Close-to-Close Volatility (CCV), Parkinson Volatility, and Garman–Klass Volatility. However, each traditional volatility estimator has limitations. CCV relies only on daily Close prices, showing low efficiency and missing intraday dynamics. Parkinson Volatility and Garman–Klass Volatility incorporate intraday ranges but assume zero price drift and no overnight gaps, assumptions frequently violated in practice [29,30,31]. To address these drawbacks, Yang and Zhang developed an estimator that integrates Open, High, Low, and Close prices, extracting market volatility more comprehensively and efficiently. Theoretical and empirical evidence confirms its superior statistical efficiency and robustness relative to traditional alternatives [32]. In the Chinese market, Zhou et al. verified that machine learning models based on Yang–Zhang Volatility achieved stable, outstanding predictive performance across indices [33]. Petneházi confirmed that the Yang–Zhang Volatility consistently outperformed counterparts that relied on traditional volatility measures. Such superiority is particularly prominent amid high market volatility and systemic stress, wherein the Yang–Zhang Volatility can precisely characterize time-varying volatility dynamics, thus providing a more reliable analytical basis for financial risk management and quantitative trading strategies [34].

### 2.3. Entropy-Based Methods in Financial Market

Forecasting accuracy depends jointly on model performance and the informational quality of input features. Traditional volatility prediction approaches rely mainly on price-based technical indicators that capture trends and momentum [35]. However, financial markets are complex nonlinear systems whose intrinsic complexity drives the dynamic evolution of asset volatility. Entropy is a canonical metric for evaluating the uncertainty of complex systems. Shannon Entropy is its most basic and widely applied form. Bentes et al. theoretically demonstrated that entropy enabled a more comprehensive characterization of the statistical structure and nonlinear dependencies embedded in financial time series data. Their cross-market comparative analysis of stock index data further validated the applicability and effectiveness of entropy-based financial market measurement [36]. As research advanced, Shannon Entropy extended from standalone volatility quantification to multidimensional evaluation of market operational states. Bengihat et al. integrated Shannon Entropy with volatility models such as Fractionally Integrated GARCH, using it not as an independent indicator but as a tool to characterize market dynamics. Their results confirmed that heterogeneous assets display distinct entropy patterns across price, return, and volatility series, reflecting cross-market differences in informational efficiency and stochastic behavior [37,38]. Derived from information theory and complex system science, Fuzzy Entropy provides a novel paradigm for volatility modeling by quantifying the complexity and irregularity of time series through the self-similarity of data patterns. Cohen et al. combined Fuzzy Entropy and Shannon Entropy as predictive features in machine learning models for industry-level stock return forecasting [39].

Despite their extensive application, Shannon Entropy and Fuzzy Entropy have inherent limitations. Shannon Entropy highly relies on the probability distribution of input data, making it susceptible to noise when series deviate from regular statistics. Fuzzy Entropy offers better noise robustness but fails to fully capture dynamic evolutionary characteristics and local patterns. To improve the feature framework, we adopt Permutation Entropy and Dispersion Entropy. Proposed by Bandt and Pompe, Permutation Entropy quantifies complexity by transforming a series into ordinal patterns and computing their probability distribution, enabling the identification of nonlinear structures [40]. Huang et al. validated its efficacy in capturing time-varying structural features [41]. Obanya et al. found that lower Permutation Entropy corresponded to a higher predictability of daily Bitcoin volatility [42]. As another method proposed by Rostaghi and Azami, Dispersion Entropy quantifies the uncertainty and complexity of time series through two core procedures: mapping original time-series data into discrete state sequences and calculating the dispersion degree of the obtained states [43]. Distinct from Permutation Entropy, Dispersion Entropy integrates the amplitude information of original time series, thereby enabling a more refined and comprehensive characterization of time series irregularity. However, the application of Dispersion Entropy in financial markets remains in its infancy, and the existing literature has not fully explored its potential value for financial market analysis and volatility forecasting.

Recently, entropy-based approaches have been extended to volatility analysis and applications. Scrucca used differential entropy estimated through Gaussian mixture models to evaluate the volatility of financial log-returns, showing that entropy provides a more robust characterization of volatility when return distributions deviate from normality [44]. At the market level, Shternshis et al. proposed a genuine estimation of Shannon Entropy for high frequency ETF time series and demonstrated that entropy-based indicators can quantify the time-varying efficiency of ETF markets [45]. In addition, Kopczewski et al. constructed entropy measures in the joint return and volume space and showed that their evolution tracks market unpredictability during global shocks, suggesting their potential as informative features in machine learning forecasting [46]. Collectively, these recent studies confirm the value of entropy indicators for financial markets. However, their application to multi-asset ETF volatility forecasting, particularly in the Chinese market, still remains limited.

### 2.4. Portfolio Construction and the Barbell Strategy

From a methodological perspective, we incorporate volatility forecasting into the Barbell Strategy, which is deliberately chosen for increasingly complex markets. By polarizing capital between two ends with heterogeneous risk drivers: a low volatility bond assets and growth-oriented equity index ETFs, the structure yields a natural hedging effect [47]. As proposed by Taleb in his work Antifragile: Things That Gain from Disorder, the Barbell Strategy can improve portfolio robustness and return potential through dynamic weight allocation between low-risk and high-risk asset pools [48]. Building on this theoretical foundation, this research innovatively integrates a volatility-driven dynamic adjustment mechanism into the conventional Barbell Strategy framework. Relying on volatility forecasting results, the proposed model dynamically calibrates asset weights and asset selection for both low-risk and high-risk asset portfolios. This optimized strategy effectively improves core investment performance metrics, including return level and risk control, providing a systematic and reliable approach to balancing downside risk protection and upside return participation across diverse market conditions.

Traditional volatility-targeting strategies aim to maintain the portfolio volatility at a predetermined target by scaling the overall risky asset exposure. Moreira et al. showed that volatility-managed portfolios that reduce exposure when volatility is high generate significant alphas [49]. However, Cederburg et al. showed that the real-time, out-of-sample combinations of scaled and unscaled portfolios generally fail to beat the original portfolios [50]. Volatility-targeting approaches stabilize portfolio volatility at a fixed level and may employ leverage when volatility falls below the target, whereas our proposed framework does not constrain volatility to a target but uses predicted Yang–Zhang Volatility as a timing signal to adjust the relative weights of the bond and stock ETF of the Barbell structure. Also, traditional volatility targeting scales a single risky portfolio against the risk-free asset, while our strategy dynamically reallocates between two structurally distinct asset classes, thereby retaining the tail-risk hedging property inherent to the Barbell allocation. In this sense, the proposed strategy integrates volatility forecasting with structural portfolio design rather than applying volatility as a risk-budgeting constraint.

Moreover, entropy-based information has recently been exploited beyond forecasting in portfolio construction. Giunta et al. showed that entropy-based portfolio strategies can deliver competitive risk-adjusted performance, particularly under heightened market risk and asset concentration [51]. These developments point to an emerging trend of entropy-driven investment design, with which the volatility-driven Barbell framework is aligned.

### 2.5. Research Gaps and Positioning

Based on the above review, several critical research gaps exist in the field of financial volatility forecasting and the practical application of entropy-based technical indicators. First, empirical research on Yang–Zhang Volatility forecasting remains insufficient, especially for multi-asset ETF portfolios. Second, while Shannon and Fuzzy Entropy have been widely applied, the use of Permutation Entropy and Dispersion Entropy in volatility prediction remains underexplored, and recent entropy-based studies have rarely been extended to ETF volatility forecasting in emerging markets such as China. Third, the existing literature focuses largely on either prediction tasks or overall volatility control, with a limited integration of volatility forecasts into executable trading strategies. To fill these deficiencies, we develop an integrated framework linking multi-window entropy-driven volatility forecasts to a dynamic Barbell Strategy, systematically examining how volatility estimators, entropy indicators, and rebalancing frequencies jointly affect forecasting and investment performance.

## 3. Materials and Methods

**Figure 1:**
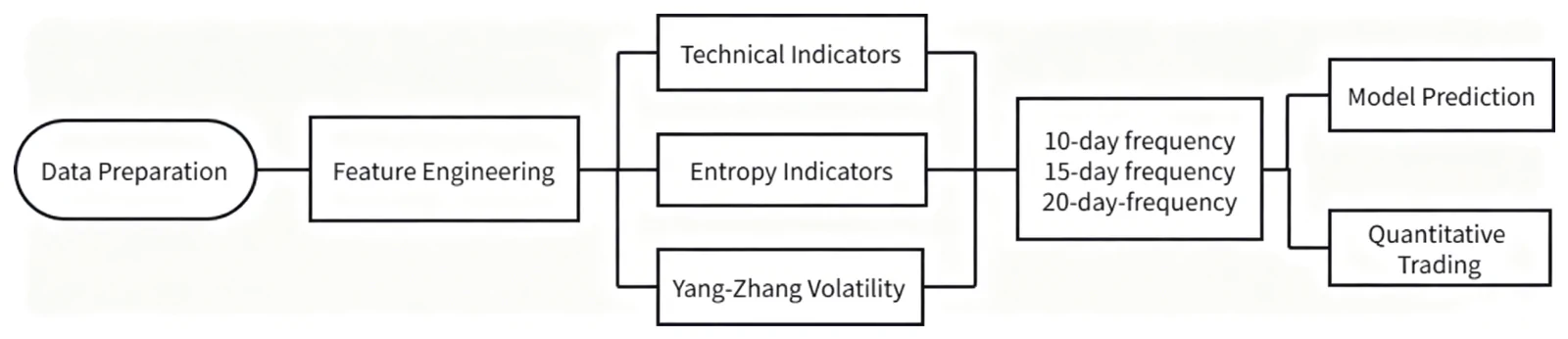
Experimental framework.

This research evaluates the predictive performance of Yang–Zhang Volatility in the Chinese market and the quantitative trading performance of a volatility-adaptive Barbell Strategy across market-capitalization segments and forecasting windows. The framework has three components. The first component covers data sources, the rolling-window experimental design for volatility prediction, and the construction of a multi-dimensional feature set integrating trend, volatility, and entropy-based technical indicators. The second component provides the details of machine learning models, such as the Random Grid Search hyperparameter optimization scheme, and the evaluation metrics. The third component describes the volatility-driven dynamic position adjustment of the Barbell Strategy based on Yang–Zhang Volatility forecasts, the backtesting framework, and the portfolio performance indicators. Figure 1 visualizes the overall framework, enabling the verification of strategy robustness across models, assets, and windows.

### 3.1. Data Preprocessing and Feature Construction

We select three core indices of the Chinese stock market as the risky-asset constituents of the Barbell Strategy: CSI 300 Index (510300), CSI 500 Index (510500), and CSI 1000 Index (512100), representing the large capitalization stocks (Large-Cap), middle capitalization stocks (Mid-Cap), and small capitalization stocks (Small-Cap). Since the 512100 ETF began trading in 2016, we use the underlying CSI 1000 index as a proxy for the pre-inception period and switch to actual ETF data thereafter. Since the ETF closely tracks its benchmark and our focus is on volatility dynamics rather than return arbitrage, this proxy has negligible impact on our findings. These three indices cover different market-capitalization segments and fully represent the domestic market. The Chinese government-bond ETF (511010) serves as the low-risk asset component. These two kinds of assets are driven by heterogeneous factors: interest-rate, credit conditions, earnings and risk appetite, which underpin the diversification benefit. All instruments feature high liquidity and superior tracking accuracy, making them feasible for both institutional and individual investors.

The sample spans 2015 to 2025, with raw data downloaded from the Investing platform [52], including Open, High, Low, and Close prices and Trading Volume. This decade covers typical market conditions and extreme events, like the 2015 turbulence, the 2018 bear market, the 2020 COVID-19 shock, and the sustained market volatility between 2022 and 2024. Such heterogeneous market environments enable a rigorous evaluation of the model’s adaptability and predictive performance across varied economic cycles. The original raw data is the total return index data, which accounts for the dividend reinvestment of constituent stocks according to official index weights, thereby capturing the full return generated by underlying assets. All data are strictly aligned with official exchange calendars, and no missing value interpolation is required. To eliminate look-ahead bias and replicate out-of-sample conditions, a rolling-window scheme is adopted for sample partitioning in this research. Specifically, the initial in-sample training window is set to 4 years, paired with a 1 year out-of-sample forecasting interval that advances annually. The walk-forward rolling partition strategy is implemented as follows: the training dataset spanning 2015–2018 is used for prediction in 2019, the training dataset spanning 2016–2019 is used for 2020 prediction, and, at last, the training dataset spanning 2021–2024 serves as the training sample for forecasting data in 2025. Following this experimental design, all forecasting and trading results are generated for the period spanning 2019 to 2025.

We further construct a feature set of more than 40 variables extracted from multidimensional time series information, covering price trends, volatility ranges, return characteristics, historical volatility, and Yang–Zhang Volatility. All feature values are computed on rolling windows to ensure consistency and avoid data leakage. To ensure the integrity of window lengths for all rolling features starting from 2015, additional historical data from 2014 is introduced to compute the indicator values for the initial rolling window. Notably, the 2014 auxiliary data is not involved in model fitting and performance evaluation, which ensures that all feature observations within the 2015–2025 sample period are derived from complete rolling calculation windows. During the model training phase, data standardization is implemented within each independent rolling window to effectively prevent future data leakage.

All preprocessing, model training, and backtesting are conducted under Python 3.13.5, with core libraries pandas 2.2.3, numpy 2.1.3, scikit-learn 1.6.1, lightgbm 4.6.0, and xgboost 3.2.0. A fixed random seed of 2026 is applied to model initialization, hyperparameter random search, and cross-validation partitioning to guarantee reproducibility. The following section introduces the feature used for machine learning construction.

#### 3.1.1. Trend, Price Range, and Volume Features

Trend-based features are fundamental to technical analysis, identifying the direction and intensity of price movements and evaluating an asset’s position within market trends [53]. Moving Average (MA) indicators are among the most common trend-tracking tools. We construct MA features over 5-day, 20-day, and 60-day windows to capture short-term, medium-term, and long-term trends, enabling the model to learn dynamic trend characteristics across diverse time scales effectively [54].

(1) $$MA_{t}\left(n\right)=\frac{1}{n}\sum_{i=t-n+1}^{t}Close_{i}.$$

The Bias indicator $Bias_{t}$ measures the deviation degree of the daily $Close_{t}$ price from its short-term and medium-term moving averages, namely, $MA_{t}\left(5\right)$ and $MA_{t}\left(20\right)$. This indicator facilitates the identification of extreme price statuses and distinct market regimes. It directly gauges the relative position of the current market price, either substantially above or below the corresponding moving average, thereby signaling potential overbought or oversold conditions in the financial market.

(2) $$Bias_{t}=\frac{Close_{t}-MA_{t}\left(n\right)}{MA_{t}\left(n\right)}.$$

To quantify the magnitude of price movement, we construct a technical feature based on daily price range information. Specifically, the indicator is defined as the ratio of the absolute daily trading range $High_{t}-Low_{t}$ to the daily $Close_{t}$ price.

Bollinger Bands constitute a prevalent technical analysis indicator that synthesizes trend and volatility information. The indicator comprises three dynamically adaptive boundaries: a middle band, an upper band, and a lower band, which adjust to accommodate time-varying market conditions [55]. In practical technical analysis, a price breakout above the upper band typically signals an overbought market status, whereas a breakdown below the lower band indicates a potential oversold condition. In our research, multiple predictive features are extracted based on Bollinger Bands:

(3) $$Middle_{t}=MA_{t}\left(20\right),$$

(4) $$Upper_{t}=Middle_{t}+2\cdot \sigma_{t}\left(Close_{t},20\right),$$

(5) $$Lower_{t}=Middle_{t}-2\cdot \sigma_{t}\left(Close_{t},20\right).$$

Trading Volume is a core technical indicator for measuring market liquidity and capital flow dynamics. The convergence and divergence relationships between price movements and Volume trends are widely adopted to evaluate the robustness and credibility of price and volatility trading signals [56]. This research constructs three daily volume changes’ features, the daily change in Volume, the 5-day MA of Volume, and the Volume ratio that quantifies the relationship between daily Volume and its 5-day MA. The calculation formula for the volume ratio is defined as follows:

(6) $$\frac{Volume_{t}}{\frac{Volume_{t}+Volume_{t-1}+\ldots +Volume_{t-4}}{5}}.$$

#### 3.1.2. Return and Momentum Features

Return rate is a direct metric for quantifying periodic asset price variations and constitutes a fundamental input for volatility estimation. We construct multiple return-based features, including daily returns, 5-day cumulative returns, and 20-day cumulative returns. To further capture the higher-order statistical moments of return distributions, which convey essential information regarding distribution characteristics and tail risk exposure. We compute return skewness and kurtosis based on a rolling 20-day window additionally.

(7) $$R_{t}\left(n\right)=\frac{Close_{t}}{Close_{t-n}}-1.$$

Return-based features are constructed to capture short-term and medium-term price momentum. The predictive efficacy of momentum strategy has been well-documented in the empirical finance literature. For example, Xu et al. demonstrated that a trading strategy integrating six month price momentum with corporate investment indicators got persistent positive returns across both bull and bear market regimes in the American stock market over a 50-year sample period, without incurring a substantial rise in portfolio risk [57]. The return-driven momentum characteristics adopted in this research enrich the multi-dimensional feature system by incorporating price trend information, thereby providing informative inputs for volatility forecasting.

Moving Average Convergence Divergence (MACD) is a classic trend-following momentum indicator conventionally applied to price time series to identify variations in trend strength, direction, and persistence. We introduce a volatility-adjusted variant of MACD to enhance cross-period comparability. Specifically, the standard MACD is computed from Closing prices using the conventional fast and slow Exponential Moving Average (EMA). The resulting MACD value is then normalized by the Average True Range (ATR), a measure of recent market volatility. This scaling ensures that the MACD feature is comparable across periods with different volatility regimes, preventing high-volatility periods from dominating the feature values. The signal line and histogram of MACD are computed following the same EMA structure as the standard MACD indicator [58].

(8) $$EMA_{t}\left(n\right)=\frac{2}{n+1}\sum_{i=0}^{t-1}{\left(1-\frac{2}{n+1}\right)}^{i}Close_{t-i},$$

(9) $$TR_{t}=max(High_{t}-Low_{t},|High_{t}-Close_{t-1}|,|Low_{t}-Close_{t-1}\left|\right)$$

(10) $$ATR_{t}\left(26\right)=EMA_{t}\left(TR,26\right)$$

(11) $$MACD\;line_{t}=\frac{EMA_{t}\left(12\right)-EMA_{t}\left(26\right)}{ATR_{t}\left(26\right)}\times 100$$

(12) $$MACD\;signal_{t}=EMA_{t}\left(MACD\;line,9\right)$$

(13) $$MACD\;hist_{t}=MACD\;line_{t}-MACD\;signal_{t}$$

#### 3.1.3. Entropy Features

Stock return time series are typically characterized by nonlinearity, non-stationarity, inherent market noise, and structural mutations. Most volatility features are developed under linear frameworks or predefined distribution assumptions, which fail to fully capture the intrinsic dynamic complexity and disordered information of financial sequences. As a powerful tool for quantifying system uncertainty and information complexity, entropy is capable of measuring the chaotic and complex properties of time series, thereby offering a novel perspective for volatility forecasting. We incorporate four representative entropy indicators to construct feature sets: Shannon Entropy, Fuzzy Entropy, Permutation Entropy, and Dispersion Entropy. Daily simple returns derived from Close prices are adopted as the basic input sequence for entropy calculation, and the return is formally defined as $\frac{Close_{t}}{Close_{t-1}}-1$. To capture the dynamic evolutionary properties of return distributions across multiple time scales, entropy-based features are computed through 10-day, 15-day, and 20-day rolling window lengths [39], which correspond to ultra-short-term and short-term dimensions for characterizing market volatility complexity and are consistent with the settings of prediction labels and portfolio rebalancing frequencies. Specifically, the entropy value at day *t* is calculated based on consecutive observations spanning from day t−n+1 to day *t*. The rolling window advances sequentially over time to generate time-varying entropy features for each trading period.

Shannon Entropy is defined based on discrete probability distributions theoretically, whereas stock returns belong to continuous random variables. Accordingly, we employ a histogram-based discretization approach to convert continuous return data into discrete observations within each rolling window. The empirical probability distribution is further estimated using sample frequency statistics. For return observations in a given rolling window, the maximum and minimum return values are defined as the upper and lower bounds of the binning range. Equal-width binning is subsequently applied to partition the overall return interval into multiple equidistant subintervals. To accommodate varying sample sizes across rolling windows and guarantee stable probability estimation as well as reliable distribution characterization, this research adopts window-specific bin numbers corresponding to different window lengths. Specifically, the 10-day rolling window is configured with 5 bins, while both the 15-day and 20-day rolling windows adopt 10 bins. Thus, the 10-day and 20-day rolling windows get an average bin sample size of 2, which guarantees a consistent baseline accuracy of probability estimation across the two window lengths. Serving as an intermediate parameter for sensitivity analysis, the 15-day windows adopts an identical bin number to that of the 20-day windows. Furthermore, the bin intervals for each window are adaptive to the fluctuation range of data dynamically, which effectively avoids the extreme sparsity issue that commonly arises with fixed bin strategies, wherein narrow local fluctuation intervals may generate empty bins with no valid samples. In addition, only bins containing non-zero sample are included in the calculation, while all empty bins are automatically excluded from the computation process. This treatment eliminates invalid disturbances from empty bins and prevents numerical anomalies of the logarithmic function in the algorithm, thereby ensuring the stability of the entropy calculation. The Shannon Entropy values are normalized by log(K), where *K* is the number of bins, to facilitate comparison across window settings with different bin counts.

Fuzzy Entropy, a time series complexity measurement algorithm proposed by Chen et al. [59], introduces a fuzzy membership function to realize the soft similarity discrimination of sequence sub-patterns. This approach effectively replaces the rigid threshold-based binary judgment adopted by Approximate Entropy and Sample Entropy. The embedding dimension is explicitly set to m=2 with a unit time delay τ=1 for time series reconstruction and phase space reconstruction. For each return subsequence within the rolling window, *m* dimensional state vectors are continuously constructed in the form of $\left[x_{i},x_{i+1},\ldots ,x_{i+m-1}\right]$, where the starting index *i* traverses all valid positions across the window. This procedure gets a total of N−m+1 reconstructed *m*-dimensional delayed vectors, with *N* denoting the length of the corresponding rolling window. Similarly, m+1-dimensional delayed vectors are constructed following identical criteria, generating N−m vectors that support the hierarchical pattern similarity comparisons in subsequent analyses. The adopted embedding dimension represents a standard for the complexity analysis of financial time series. This setting enables the effective capture of local market fluctuation patterns. The similarity between vectors is measured using the Chebyshev distance, and the fuzzy membership function takes the form $e^{-{\left(\frac{d}{r}\right)}^{n}}$, where d is the Chebyshev distance, r is the similarity tolerance, calculated as 0.2 times the rolling window Standard Deviation, and n=2 is the fuzzy weight index, corresponding to a Gaussian-type membership function. Self-matches i=j are excluded from the similarity calculation.

Permutation Entropy is a nonlinear dynamic metric that quantifies the complexity and irregularity of time series by exploiting the ordinal sorting relationships among sequential observations. To capture the multi-scale and short-term fluctuation complexity of stock return series, we adopt a rolling-window scheme to generate time-varying permutation entropy sequences. In all window-based calculations, the embedding dimension is fixed at 3. This prevalent parameter configuration is widely validated in financial time series analysis, as it effectively characterizes typical short-term market fluctuation patterns, including sustained upward trends, continuous downward trends, and volatile market states. Additionally, the time delay parameter is set to 1. For daily return data, a unit time delay preserves the temporal continuity of adjacent price variations and guarantees the integrity of short-term dynamic market information. Tied return values are handled using the ordinal ranking from stable sorting, where equal values are assigned ranks based on their temporal order of appearance. The Permutation Entropy is normalized by log(m!) to ensure values fall within the [0,1] interval, facilitating interpretation and comparison across different parameter settings. A value of 0 indicates a perfectly regular sequence, while a value of 1 indicates maximum complexity where all ordinal patterns are equally likely.

Proposed by Rostaghi and Azami, Dispersion Entropy quantifies the irregularity and dynamic complexity of time series by evaluating the distribution uniformity of discrete amplitude patterns. Compared with other entropy algorithms, Dispersion Entropy exhibits computational efficiency and estimation stability for short time series [43], rendering it highly applicable for multi-scale complexity analysis of financial data. For return subsequences within each rolling window of length *n*, we apply the Normal Cumulative Distribution Function to map raw return observations to the interval [0, 1]. The Mean and Standard Deviation of in-window samples are adopted as the parameters of the Normal Distribution for mapping, which mitigates estimation bias of Dispersion Entropy induced by extreme return values. The number of classes *c* is fixed at 6. Continuous normalized values within [0, 1] are transformed into integer symbolic sequences ranging from 1 to 6 through linear scaling and rounding operations. The embedding dimension *m* is set to 3 and the time delay τ is set to 1. Accordingly, each dispersion mode is constituted by three consecutive symbolic values within the rolling window, generating a total of $c^{m}=216$ potential mode states in the mode space. This parameter setting balances the richness of spatial mode representation and the statistical validity of finite short-window samples, representing a widely adopted configuration for entropy feature extraction in financial time series analysis. Following symbolic discretization, the frequency of each dispersion mode within the window is statistically quantified, and the corresponding window-specific Dispersion Entropy is calculated using the standard Shannon Entropy formula. Regarding zero-return processing, zero-return and non-zero-return samples undergo identical normalization, discretization, and mode counting pipelines, which guarantees consistent computational logic and preserves the full information content of the original return sequences. In terms of boundary value handling, samples with equal amplitude values during discretization are consistently assigned to the same class for mode statistics without random perturbation or sorting adjustment. Meanwhile, out-of-bound values exceeding the valid interval are uniformly categorized into the nearest valid class to prevent undefined symbolic outputs and ensure stability. With c=6 classes and embedding dimension m=3, the dispersion entropy state space comprises 216 possible patterns, while rolling windows of 10 to 20 days yield a few observed patterns. This limited pattern coverage is a well-known property of Dispersion Entropy when applied to very short time series, and the resulting estimates are necessarily noisier than those from longer windows. However, Dispersion Entropy serves as a predictive feature rather than as a precise measure of complexity. What matters for our purpose is whether the feature carries incremental information useful for forecasting.

#### 3.1.4. Historical Volatility and Yang–Zhang Volatility

We also construct dynamic volatility characteristics such as Historical Volatility (HV), defined as the standard deviation of historical log returns and offering a direct, intuitive estimate of price fluctuations over a given horizon [60]. HV is computed from daily log returns over 5-day, 20-day, and 60-day windows. The corresponding calculation formula for an n-day rolling window is defined as follows:

(14) $$HV_{t}\left(n\right)=\sigma_{t}\left(R_{t}\left(1\right),R_{t-1}\left(1\right),\ldots ,R_{t-n+1}\left(1\right)\right).$$

We further construct volatility ratio indicators to characterize the time-varying term structure of volatility and identify shifting regimes. Specifically, two types of volatility ratios are adopted: the ratio of 5-day HV to 20-day HV, and the ratio of 20-day HV to 60-day HV. These complementary metrics reflect the acceleration or deceleration of market uncertainty and enable a fine-grained depiction of evolving volatility conditions.

Unlike HV, which derives solely from Close prices, Yang–Zhang Volatility is a more efficient estimator that integrates Open, High, Low, and Close daily prices. Using full-specification price data substantially reduces estimation variance and yields statistically robust measurements. The future one-day-ahead Yang–Zhang Volatility is defined as the predictive target $y_{t}$ of the forecasting framework, estimated over 10-day, 15-day, and 20-day rolling windows corresponding to short-term and medium-term volatility horizons. Furthermore, lagged Yang–Zhang Volatility series at multiple time lags 1, 2, 3, 5, and 10 days, together with their 5-day and 10-day MA trends, are constructed as predictive features. The formal calculation formula over a window of n day Yang–Zhang Volatility is specified as follows:

(15) $$y_{t}=\sigma_{YZ,t+1};$$

(16) $$\sigma_{YZ,t}^{2}=\sigma_{o,t}^{2}+\alpha \sigma_{c,t}^{2}+\left(1-\alpha \right)\sigma_{rs,t}^{2};$$

(17) $$\alpha =\frac{0.34}{1.34+\frac{n+1}{n-1}};$$

(18) $$\sigma_{o,t}^{2}=\frac{1}{n-1}\sum_{k=0}^{n-1}{\left(\ln\frac{Open_{t-k}}{Close_{t-k-1}}-\frac{1}{n}\sum_{k=0}^{n-1}\ln\frac{Open_{t-k}}{Close_{t-k-1}}\right)}^{2},$$

(19) $$\sigma_{c,t}^{2}=\frac{1}{n-1}\sum_{k=0}^{n-1}{\left(\ln\frac{Close_{t-k}}{Open_{t-k}}-\frac{1}{n}\sum_{k=0}^{n-1}\ln\frac{Close_{t-k}}{Open_{t-k}}\right)}^{2},$$

(20) $$\sigma_{rs,t}^{2}=\frac{1}{n}\sum_{k=0}^{n-1}\ln\left(\frac{High_{t-k}}{Close_{t-k}}\right)\ln\left(\frac{High_{t-k}}{Open_{t-k}}\right)+\ln\left(\frac{Low_{t-k}}{Close_{t-k}}\right)\ln\left(\frac{Low_{t-k}}{Open_{t-k}}\right).$$

### 3.2. Model Construction Process

To evaluate modeling approaches systematically, we use seven machine learning models across three categories: Decision Tree, bagging ensemble, and boosting ensemble methods. This selection enables comprehensive comparison of generalization and predictive accuracy across algorithmic structures. Random Forest and Extra Trees are representative bagging-based methods that improve performance mainly by reducing model variance. And AdaBoost, Gradient Boosting, XGBoost, and LightGBM belong to the boosting ensemble methods. These algorithms construct ensemble models in a sequential manner, where each new trained learner iteratively corrects the residual errors generated by the cumulative outputs of previous base models. The Decision Tree serves as the fundamental component, providing a reliable benchmark and enhancing the interpretability of the ensemble framework.

In the rolling-window training procedure, hyperparameters are optimized by Randomized Grid Search combined with 5-fold time-series cross-validation, minimizing the Root Mean Squared Error (RMSE) across validation folds. Compared with Grid Search, this approach is more computationally efficient and yields more stable generalization in high-dimensional parameter spaces. The candidate domains are referenced from existing research and adapted to this setting [28,38]. The 5-fold time-series cross-validation used for hyperparameter tuning strictly adheres to the walk-forward splitting principle. Specifically, in each fold, the training set corresponds to the earlier time period, and the validation set consists of subsequent temporal observations, with no temporal overlap between the two subsets. The detailed hyperparameter search space for each machine learning model is presented below:

1. AdaBoost: n_estimators (50, 150, 200), learning_rate (0.01, 0.05, 0.1, 0.5).
2. Decision Tree: max_depth (2, 3, 4, 5, 6), min_samples_leaf (3, 5, 7, 9), min_samples_split (2, 3, 4, 5).
3. Extra Trees: n_estimators (50, 100, 200, 300), max_depth (5, 10, None), min_samples_split (2, 5, 10), min_samples_leaf (2, 4, 6), max_features (1.0, sqrt, log2), bootstrap (True, False), criterion (squared_error, absolute_error, friedman_mse).
4. Gradient Boosting: n_estimators (50, 100, 150, 200), learning_rate (0.01, 0.1, 0.2), max_depth (3, 4, 5, 6), min_samples_split: (2, 5, 10), min_samples_leaf: (2, 4, 6), max_features (sqrt, log2, None).
5. LightGBM: n_estimators (50, 100, 150, 200), learning_rate: (0.01, 0.1, 0.5), max_depth: (3, 5, 7, −1), num_leaves (10, 20, 30), min_child_samples: (20, 30, 50, 100), reg_alpha (0, 0.1, 0.5, 1.0), reg_lambda (0, 0.1, 0.5, 1.0).
6. Random Forest: n_estimators (50, 100, 150), max_depth (10, 25, None), min_samples_split (2, 5, 10), min_samples_leaf (2, 4, 6), max_features (sqrt, log2).
7. XGBoost: n_estimators (50, 100, 150, 200), max_depth (3, 5, 7, 9), learning_rate (0.001, 0.01, 0.1, 0.2), subsample (0.7, 0.8, 0.9, 1.0), gamma (0, 0.1, 0.2, 0.5), reg_alpha (0, 0.1, 0.5, 1.0), reg_lambda (0, 0.1, 0.5, 1.0).

To prevent label leakage at data boundaries, the last observation of each training fold is removed, since its corresponding target requires data from the first day of the validation fold. And all preprocessing steps are performed independently within each cross-validation fold, using only training-fold data to fit the scaler.

To quantitatively assess the predictive performance of the Yang–Zhang Volatility forecasting framework, we use three standard regression metrics: the R Squared, Mean Absolute Error (MAE), and Mean Squared Error (MSE). Under the standard regression, the R Squared ranges from 0 to 1. For out-of-sample R Squared, a negative value indicates poor performance. MAE and MSE measure the average absolute and squared deviations between predicted and actual volatility observations, respectively. For these two metrics, values closer to 0 signify superior model predictive accuracy. The formal mathematical definitions of these evaluation criteria are presented as follows:

(21) $$R\;Squared=1-\frac{\sum_{t=1}^{n}{\left(y_{t}-{\hat{y}}_{t}\right)}^{2}}{\sum_{t=1}^{n}{\left(y_{t}-\bar{y}\right)}^{2}};$$

(22) $$MAE=\frac{1}{n}\sum_{t=1}^{n}\left|y_{t}-{\hat{y}}_{t}\right|;$$

(23) $$MSE=\frac{1}{n}\sum_{t=1}^{n}{\left(y_{t}-{\hat{y}}_{t}\right)}^{2}.$$

### 3.3. Quantitative Trading Construction

In the trading strategy over 2019–2025, the predicted Yang–Zhang Volatility generated by the machine learning models is incorporated into the dynamic position adjustment module of the Barbell Strategy, forming a risk-adaptive asset allocation that optimizes the trade-off between return acquisition and downside risk control. The Barbell Strategy comprises two assets: the high-risk segment holds stock index ETFs across Large-Cap, Mid-Cap, and Small-Cap styles, capturing returns across market segments, while the bond ETF occupies the low-risk segment as a safe asset that stabilizes portfolio volatility. The Yang–Zhang Volatility 10-day, 15-day, and 20-day prediction windows correspond to three rebalancing frequencies for dynamic position adjustment. The core strategy mechanism is designed as follows: the dynamic weight of stock index ETFs $w_{stock,t}$ is endogenously determined by the predicted Yang–Zhang Volatility. Stock positions are adjusted inversely in response to time-varying market risk. The detailed formula for position weighting calculation is defined below:

(24) $$w_{stock,t}=\frac{50}{1+X_{t-1}},$$

(25) $$X_{t}=\frac{{\hat{\sigma }}_{YZ,t+1}}{MA_{t}\left(\sigma_{YZ,t},20\right)}-1.$$

The backtest follows a strictly chronological execution sequence. On day *t*, after all data for day *t* are available, we compute all features and generate the t+1 volatility forecast. The portfolio weight is then updated based on this forecast, and rebalancing is executed on day t+1. In the proposed framework, $\sigma_{YZ,t+1}$ denotes the next-day Yang–Zhang Volatility predicted by machine learning models. For $\sigma_{YZ,t+1}$, three distinct prediction targets are defined based on Yang–Zhang Volatility estimates with rolling window lengths of 10-day, 15-day, and 20-day. $MA_{t}\left(\sigma_{YZ,t},20\right)$ represents the moving average of historical Yang–Zhang Volatility up to time step *t*, which captures the prevailing normal fluctuation trend of the market. The relative ratio between predicted volatility and historically smoothed volatility quantifies the degree to which current market risk deviates from its regular level. The Barbell Strategy adopts a neutral benchmark allocation of 50% equity assets and 50% treasury bond assets. When the expected market volatility rises, $X_{t}$ yields a positive value. Correspondingly, the strategic equity weight is reduced and bond holdings are increased to constrain overall portfolio downside risk. Conversely, a decline in expected volatility generates a negative $X_{t}$, which increases stock exposure and enhances the return elasticity of the overall portfolio. Furthermore, considering the practical constraints of normal trading accounts that prohibit leveraged trading and bond short selling, the stock weight in this strategy is bounded within a feasible range of 0% to 100%. In extreme low-volatility market scenarios where the theoretically calculated equity weight exceeds 100%, an upper-bound constraint is activated to fix the equity allocation at 100%, with all capital invested in stock index ETFs without leverage. This boundary restriction mechanism ensures the practical feasibility and compliance of the strategy under extreme market conditions, aligning the algorithmic trading logic with real-world institutional and retail trading constraints.

To replicate market trading conditions, a fixed transaction cost of 0.03% is imposed for each rebalancing trade, with an initial capital of 1,000,000 CNY. Because the strategy is long-term and low-frequency, turnover is limited, and the fixed 0.03% cost is conservative relative to typical trading costs for liquid ETFs. Multiple metrics are used to evaluate strategy performance. Return is the total profit generated over the entire backtesting period, and Annualized Return standardizes performance over time using 252 annual trading days, with higher values indicating better profitability. *N* denotes the total number of trading days within the backtest sample period.

(26) $$Return=\frac{Asset_{end}}{Asset_{initial}}-1,$$

(27) $$Annualized\;Return={\left(\frac{Asset_{end}}{Asset_{initial}}\right)}^{\frac{252}{N}}-1.$$

Maximum Drawdown captures the worst case downside loss of the portfolio and serves as a key indicator of downside risk exposure, with lower values indicating stronger loss containment. The Calmar Ratio, defined as Annualized Return divided by the absolute value of Maximum Drawdown, links return to downside risk, with higher values indicating better risk-adjusted profitability and robustness.

## 4. Experimental Results

This section presents the results of Yang–Zhang Volatility forecasting and the corresponding Barbell Strategy, evaluating performance across entropy features, prediction windows, and machine learning models. The overall analysis is divided into two core parts: the predictive efficacy of the forecasting models and the backtesting performance of the proposed Barbell Strategy. The framework covers three ETFs, seven machine learning models, four entropy features, and three rolling windows. Forecasting accuracy is measured by the coefficient of R Squared, MAE, and MSE quantitatively. Furthermore, Return, Annualized Return, Maximum Drawdown, and Calmar Ratio are adopted to assess the practical performance of the constructed trading strategies. All results are reported as Mean ± Standard Deviation, which are statistically calculated based on rolling out-of-sample test data. Box plots and scatter plots are further utilized to visualize the distribution properties and stability of the predictive and trading outcomes. This multi-dimensional empirical analysis provides reliable evidence for the design of quantitative forecasting models and the optimization of adaptive investment strategies. All the results are calculated based on the data from 2019 to 2025.

To verify whether entropy-based variables extract information, we conduct comparative analyses based on the HAR model. The benchmark HAR feature set incorporates daily, weekly, and monthly realized volatility. On this basis, four entropy indicators are added to form an extended HAR framework. As shown in Table 1, the inclusion of entropy variables consistently improves the explanatory power of the HAR model across all market index samples, although the error differences are not significant. In particular, the R Squared for the Large-Cap ETF rises from 0.140 to 0.147, with comparable gains for Mid-Cap and Small-Cap ETFs. These results show that entropy metrics serve as informative features for volatility forecasting. We therefore adopt them as core inputs in the machine learning models and the quantitative trading framework.

### 4.1. Volatility Forecasting Results

**Figure 2:**
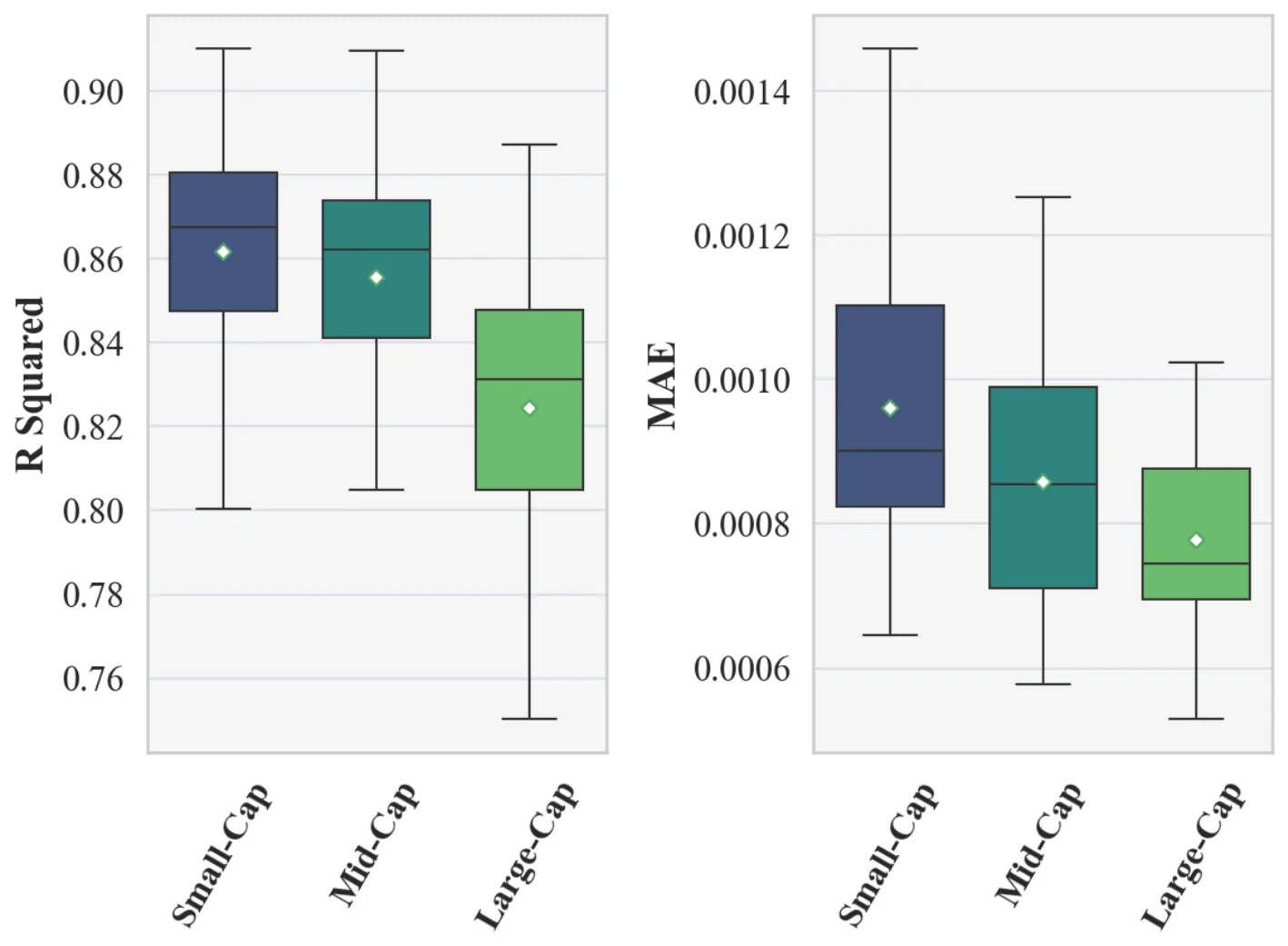
Comparisons of regression results based on different ETFs.

The 252 parameter configurations share the same underlying data period and are not independent observations. We therefore focus on the consistency of results across configurations and the economic magnitude of performance differences. We conduct multi-dimensional comparative experiments across market capitalization segments, models, feature combinations, and prediction frequencies. Baseline models exclude entropy features, and the forecasting performance for different ETFs is evaluated to quantify the incremental contribution of entropy in the prediction. The results for Large-Cap, Mid-Cap, and Small-Cap ETFs are presented in Table 2 and Figure 2. Inter-group comparisons of the ablation experiments reveal that adding entropy features yields consistent and substantial predictive improvements across all ETFs, verifying their universal effectiveness. Notably, the Small-Cap ETF achieves the most pronounced gain, suggesting that entropy features provide the largest marginal benefit for Yang–Zhang Volatility prediction in the Small-Cap market.

Across all entropy-augmented models, the Small-Cap ETF exhibits the highest predictive R Squared and the Large-Cap ETF exhibits the lowest. This reflects the ability of entropy-augmented machine learning models to capture the complex volatility dynamics of Chinese Small-Cap stocks, whose frequent price oscillations and strong nonlinearity are well-quantified by entropy-based complexity indicators. In terms of error metrics, both MAE and MSE increase monotonically as market capitalization declines, consistent with the intrinsically higher volatility of Small-Cap assets and their correspondingly larger prediction errors. Nevertheless, Small-Cap portfolios still attain the highest R Squared despite larger errors, indicating that the model has stronger explanatory power for the time-varying volatility of Small-Cap assets.

The box plots in Figure 2 corroborate these findings: the Small-Cap ETF has the highest median R Squared and the most concentrated distribution, demonstrating stable, robust explanatory power, whereas it also shows the highest median MAE, with the Large-Cap ETF showing the lowest and most compact error distribution, consistent with the standard deviations in Table 2. This pattern reflects the market characteristics of distinct capitalization scales: Large-Cap components generally exhibit lower market noise and hence lower prediction errors. Although models capture the fundamental volatility trends of Small-Cap assets effectively, substantial random noise in short-term price fluctuations inevitably raises their errors. Nonetheless, the consistently high R Squared confirms that the framework maintains superior relative predictive performance for Small-Cap volatility.

To evaluate the statistical significance of R Squared differences across the Large-Cap, Mid-Cap, and Small-Cap groups, we use paired-sample *t*-tests for pairwise comparisons. All experimental settings are strictly matched across groups to eliminate confounding effects from feature specifications, trading frequencies, and models. Each pairwise comparison is implemented under identical conditions, with paired samples based on consistent features, frequencies, and prediction models. The *t*-tests indicate statistically significant differences among all paired combinations, with all *p*-values lower than 0.001, revealing a clear hierarchy: Small-Cap has the highest R Squared, followed sequentially by Mid-Cap and Large-Cap.

**Figure 3:**
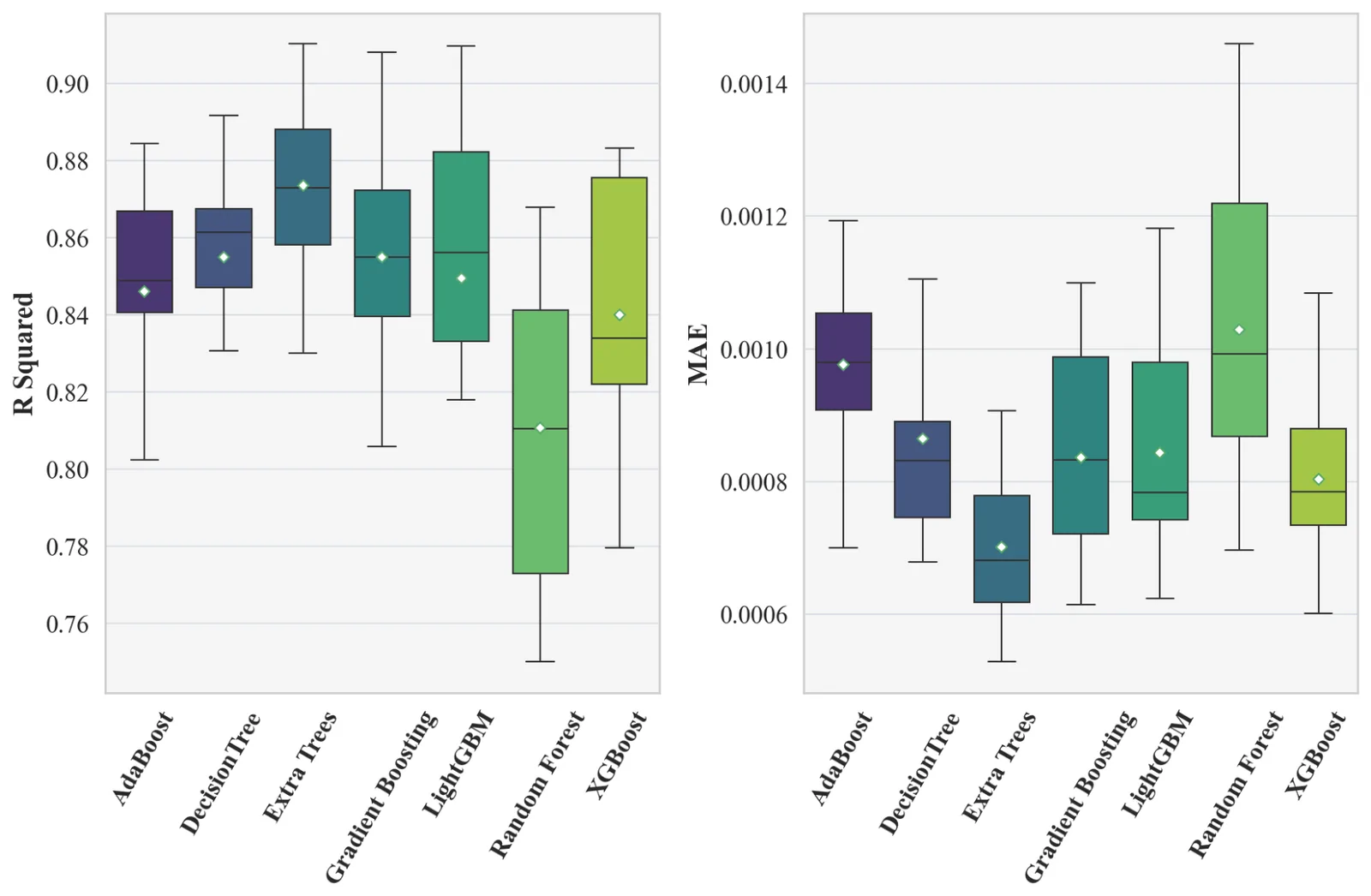
Comparisons of regression results based on different models.

With the trading ETF, entropy type, and rolling window strictly fixed as controlled variables, we compare overall model performance. Table 3 shows that the Extra Trees model achieves the best performance across all evaluation metrics, with Decision Tree and Gradient Boosting delivering competitive outcomes, but Random Forest performs weakest. The box plots in Figure 3 provide visual evidence: Extra Trees has the highest median R Squared, indicating outstanding and highly stable explanatory power, and also maintains the lowest median error with a compact distribution, confirming superior accuracy. Collectively, these results establish Extra Trees as the most effective and reliable algorithm for Yang–Zhang volatility forecasting under our framework. One-way Analysis of Variance (ANOVA) reveals statistically significant and stable differences in R Squared across model architectures with p<0.001, confirming distinct performance disparities among models.

Table 4 presents the performance of the four entropy indicators under the 10-day, 15-day, and 20-day windows. These results show that window size significantly affects forecasting performance. For all entropy-based feature sets, extending the window from 10 to 20 days raises R Squared and makes MAE and MSE lower. The average R Squared of the four indicators rises from about 0.82 in the 10-day window to 0.84 ain the 15-day window and 0.87 in the 20-day window. This improvement indicates that longer windows integrate smoother trends over an extended range, enhancing the model’s ability to capture and forecast volatility dynamics. Among the four metrics, Shannon Entropy achieves the best performance under the 20-day window, with Dispersion Entropy delivering comparable, slightly inferior results. The gaps across indicators are marginal for the 10-day and 15-day windows. Shannon Entropy’s superiority under longer windows stems from its ability to quantify the overall uncertainty and information in a series, while Dispersion Entropy’s robust performance reflects a design that combines amplitude and ordinal-pattern information, providing strong noise resistance and stable feature extraction.

### 4.2. Barbell Strategy Trading Results

To validate the practical value of the forecasting framework integrated with market capitalization characteristics, multi-entropy features, and diverse prediction windows, we construct and backtest a Barbell Strategy over 2019–2025. The investment comprises Large-Cap, Mid-Cap, and Small-Cap ETFs, and backtesting systematically compares benchmark strategies with the machine learning-driven dynamic rebalancing strategies. To ensure information-frequency consistency, the prediction window and portfolio rebalancing frequency are set to the same time scale, so that signal generation and portfolio decisions operate on the same temporal resolution. Investors who generate volatility signals at a particular time scale typically rebalance their portfolios at a comparable frequency rather than mixing disparate time horizons. The benchmark portfolio set consists of three pure stock portfolios and three stock-bond allocation portfolios built following the classic Markowitz portfolio theory [61]. The dynamic group adopts 10-day, 15-day, and 20-day rebalancing frequencies for the Barbell Strategy. Detailed results are presented in Table 5 and Table 6.

Table 5 summarizes benchmark performance. Pure stock portfolios have higher absolute returns than stock-bond portfolios but carry substantially elevated downside risk. Among the three capitalization indices, Mid-Cap achieves the best performance with an 80.7% Return and 9.2% Annualized Return, while Large-Cap shows the lowest; Mid-Cap also has the lowest Maximum Drawdown among the stock assets and the highest Calmar Ratio of 0.22, well above Large-Cap and Small-Cap. Adding bonds significantly reduces the Maximum Drawdown for all three capitalization portfolios. For example, Mid-Cap falls from 41.8% to 26.9%, but, because fixed-income assets earn less, absolute profitability declines correspondingly: Annualized Returns for Large-Cap, Mid-Cap, and Small-Cap fall to 5.8%, 7.4%, and 6.9%. All stock-bond portfolios deliver higher Calmar Ratios than their pure stock portfolios, with Mid-Cap achieving the highest Calmar Ratio of 0.27 among all benchmarks, confirming that asset allocation improves risk–return performance by balancing return acquisition and downside risk exposure.

As reported in Table 6, after controlling for entropy features and machine learning models, the dynamic rebalancing Barbell Strategy shows clear advantages in downside risk control and risk-adjusted returns, though performance varies substantially across rebalancing frequencies and capitalization styles. The 10-day strategy is the weakest across all three portfolios, with Annualized Returns below 4% and Calmar Ratios from 0.12 to 0.16, well below the stock-bond benchmarks. Its relatively high Standard Deviation suggests that excessively frequent rebalancing is highly susceptible to short term noise, undermining execution stability. The 15-day rebalancing scheme produces return profiles most comparable to the benchmarks, with average Annualized Returns of 5.4%, 6.2%, and 6.1% for Large-Cap, Mid-Cap, and Small-Cap, marginally below their benchmarks. Nevertheless, the 15-day strategy achieves lower Maximum Drawdowns than the benchmarks in Table 5. The 20-day strategy yields the best risk–return profile, with all three portfolios having higher Calmar Ratios than their benchmarks. Mid-Cap maintains the best trade-off, followed by Small-Cap and Large-Cap. This consistent performance hierarchy indicates that the inherent risk–return characteristics of capitalization styles are stable and not substantially altered by dynamic machine learning rebalancing. ANOVA examining Annualized Return across rebalancing frequencies and trading ETFs confirms significant return disparities: the 15-day strategy achieves the highest return, and strategies built on Mid-Cap and Small-Cap perform better. For risk-averse investors, the Mid-Cap Barbell Strategy with 20-day rebalancing is advisable; investors pursuing higher returns are recommended the Mid-Cap or Small-Cap strategies with 15-day rebalancing.

**Figure 4:**
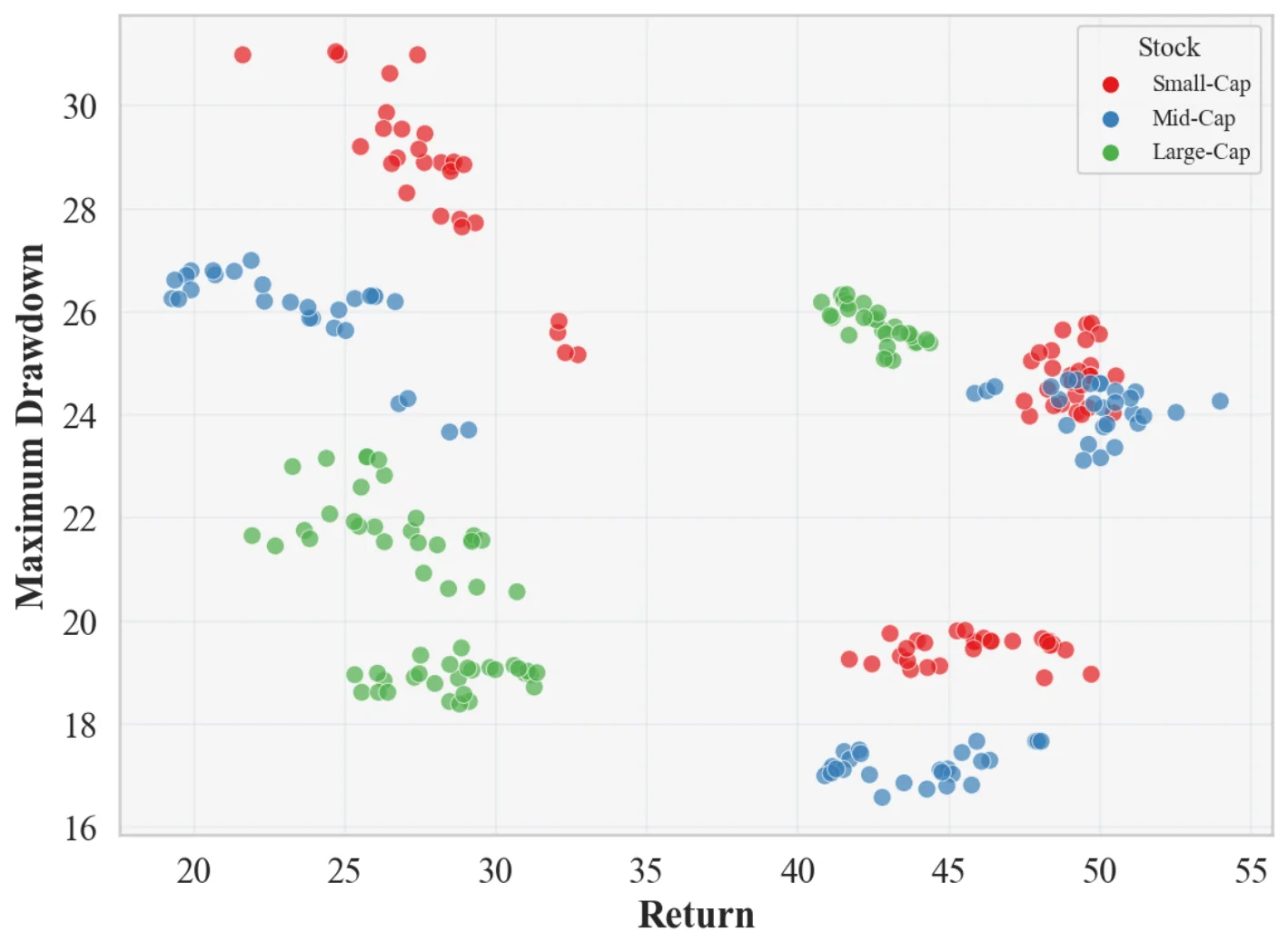
Comparisons of return and Maximum Drawdown based on different ETFs.

The scatter plot in Figure 4 illustrates the interactive relationships among market capitalization, return, and Maximum Drawdown. Higher returns are generally accompanied by higher Maximum Drawdown levels. At identical return levels, the Small-Cap points lie above those of Mid-Cap, showing that Small-Cap portfolios incur substantially higher downside risk to achieve equivalent returns. Furthermore, considerable data dispersion is evident within each market capitalization group. Such variability indicates that, apart from the fundamental impact of market capitalization characteristics, endogenous experimental designs, including machine learning algorithm selection, entropy feature adoption, and prediction window specification, exert significant joint effects on the ultimate trading performance.

After controlling for capitalization, entropy features, and rebalancing frequencies, Table 7 presents the backtesting performance of the Barbell Strategy driven by seven machine learning models. Random Forest achieves the best overall trading performance, with an average Return of 39.4% and the lowest Maximum Drawdown, highlighting its capital-preservation capability during downturns. LightGBM and Gradient Boosting also show relatively low drawdowns, whereas Decision Tree suffers the highest Maximum Drawdown and weakest risk control capacity. In comprehensive risk–return terms, Random Forest produces the highest Calmar Ratio, making it the most effective algorithm for the trading framework. Notably, the cross-algorithm ranking derived from backtesting substantially diverges from the forecasting ranking: Gradient Boosting, which forecasts accurately, ranks lowest in trading profitability, whereas Random Forest, with lower forecasting accuracy, dominates in risk-adjusted returns. This discrepancy indicates that superior predictive accuracy does not necessarily translate into better trading performance. The profitability of quantitative strategies depends not only on forecasting precision but also on practical factors, such as rebalancing frequency and embedded risk control. Machine learning forecasting minimizes numerical fitting errors of target volatility values, whereas trading profitability is primarily determined by price movements and the loss ratio, with a relatively higher tolerance for estimation errors. The Barbell Strategy relies on dual-end asset allocation to hedge market volatility, and Random Forest generates conservative fitting outputs for extreme market samples, making it more compatible with the strategy’s risk-oriented design. Furthermore, higher trading frequency makes strategies more sensitive to minor fluctuations, triggering excessive signals and elevating transaction costs. Such frequent rebalancing erodes returns and undermines performance.

**Figure 5:**
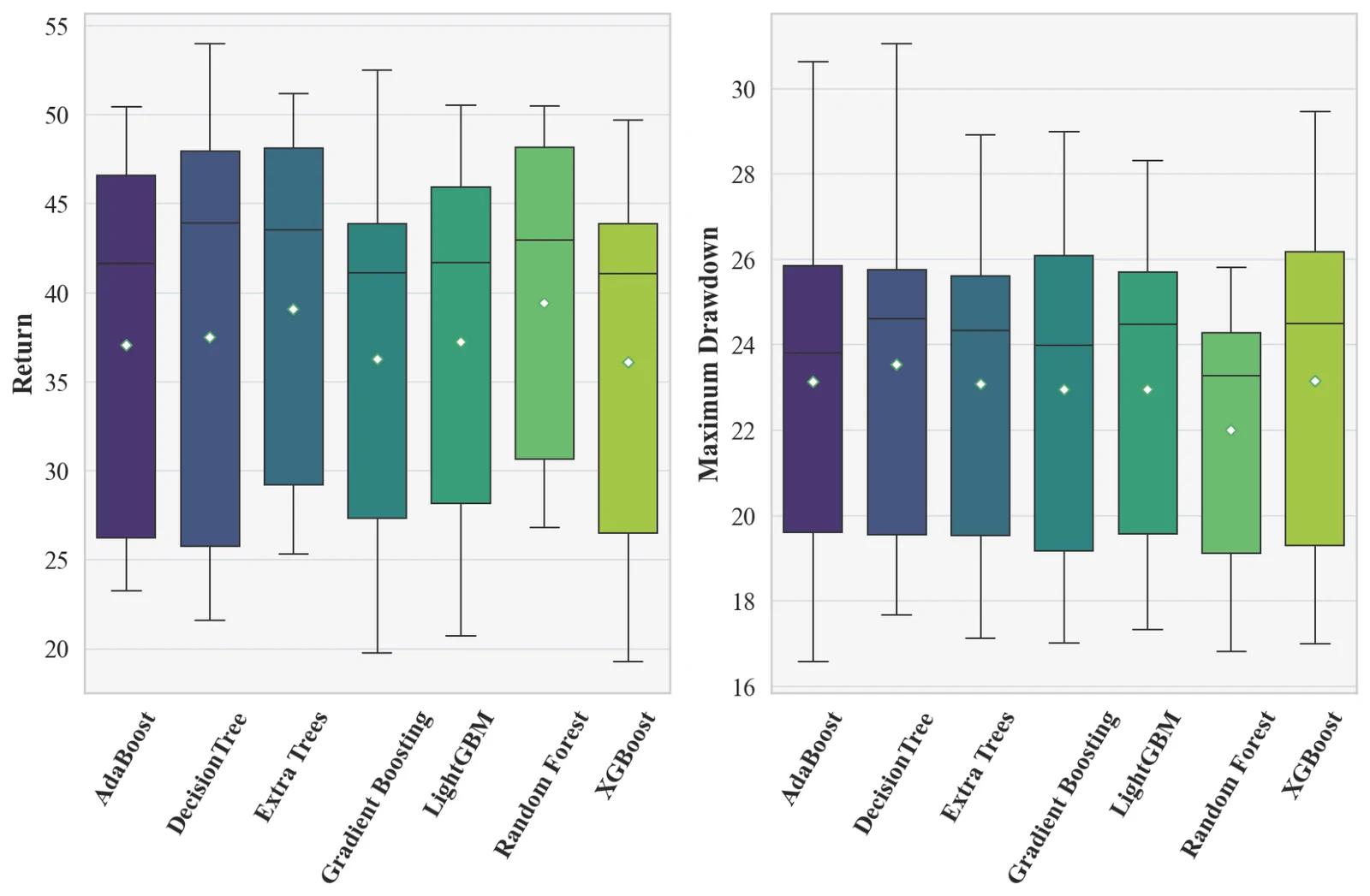
Comparisons of trading results based on different models.

Figure 5 provides the distribution of trading performance across models through box plots. Random Forest and Extra Trees yield the highest median returns, showing that these bagging-based algorithms deliver superior, stable average profitability across sample intervals. By contrast, XGBoost and Gradient Boosting show lower median returns with wider distributions, indicating volatile performance, consistent with the sensitivity of boosting frameworks to noise and their susceptibility to overfitting. For Maximum Drawdown, Random Forest exhibits the lowest median and narrowest range, confirming outstanding downside-risk control. Overall, bagging ensembles dominate in both return acquisition and risk management, with high profitability and low portfolio volatility. This evidence further shows that superior forecasting accuracy does not automatically confer trading advantages. Instead, excessive fitting to market noise undermines out-of-sample trading stability. However, contrary to the differential predictive performance across models, ANOVA reveals no statistically significant differences in Annualized Returns among models. Investors are therefore advised to evaluate model characteristics beyond predictive efficacy, including risk control and computational efficiency. For predictive accuracy, Extra Trees is preferable, while Random Forest achieves superior backtesting performance. Trading frequency and target selection influence outcomes more than the choice of machine learning models.

Table 8 shows the trading performance of strategies based on the four entropy indicators under the three rebalancing frequencies. The rebalancing interval substantially influences overall profitability and risk. The 15-day frequency yields the highest return, averaging about 47% across all entropy features. The 20-day frequency generates a moderate average of around 39%, and the 10-day frequency produces the lowest, merely 25%. This pattern deviates distinctly from the forecasting results, where accuracy improves monotonically with longer windows. In terms of downside risk, Maximum Drawdown decreases as the rebalancing cycle lengthens. It peaks near 25% at 10-day frequency, declines to about 24% at 15-day frequency, and reaches a minimum of 18% at 20-day frequency. This trend shows that strategies relying on longer-term forecasts and less frequent rebalancing achieve superior capital preservation in adverse markets. The Calmar Ratio is used to assess risk-adjusted performance. The 15-day strategy delivers higher returns, whereas the 20-day strategy achieves a better Calmar Ratio, indicating that the substantial reduction in drawdowns fully offsets the moderate decline in Annualized Returns. The 10-day strategy performs worst across all metrics due to a combination of excessive downside risk and inferior profitability. These findings underscore the need for multi-dimensional trade-offs among predictive accuracy, signal timeliness, and transaction costs in quantitative strategy design. Overemphasizing a single indicator, such as pure forecasting accuracy or absolute return, leads to suboptimal out-of-sample performance. In our research, the 20-day frequency is verified as the optimal configuration, effectively balancing return acquisition, risk exposure, and trading frictions.

Among the four entropy indicators, Dispersion Entropy achieves the best return performance under the 15-day frequency, closely followed by Permutation Entropy and Fuzzy Entropy, with Shannon Entropy producing relatively lower returns. The disparities become negligible at the 20-day frequency, with return gaps within 0.3%. These results suggest that entropy metrics integrating both amplitude and ordinal pattern information are more effective in guiding trading decisions at the 15-day rebalancing interval. And there is no significant difference among entropy indicators under low-frequency strategies, resulting in highly homogeneous performance across alternative entropy feature specifications.

**Figure 6:**
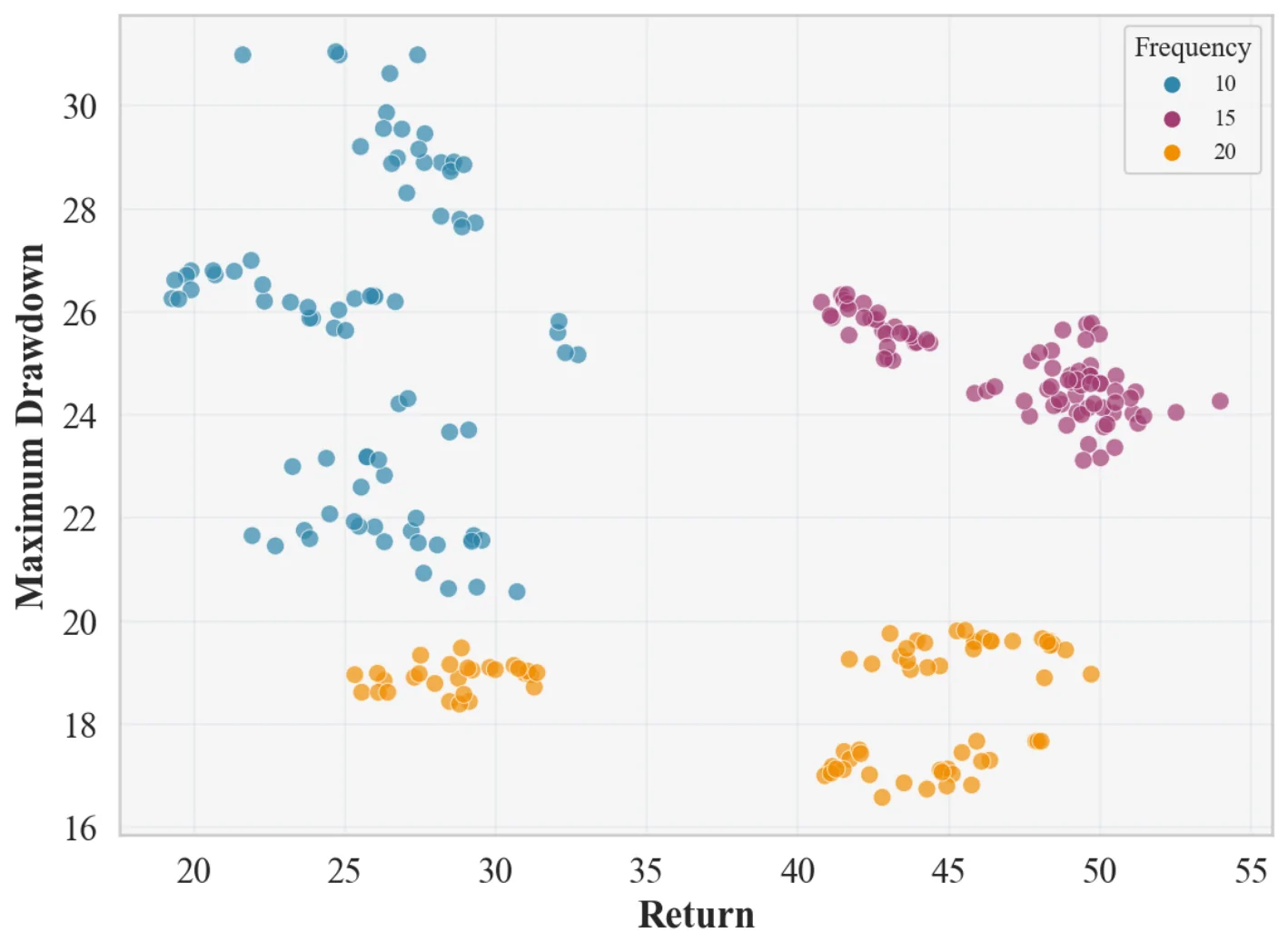
Comparisons of returns and Maximum Drawdown based on different frequencies.

Figure 6 visualizes the joint relationship among rebalancing frequency, return, and Maximum Drawdown. A clear clustering pattern emerges: the 15-day data points concentrate in the high-return, medium-risk region; the 20-day samples cluster in the medium-return, low-risk region; and the 10-day samples concentrate in the low-return, high-risk region. This corroborates that the 20-day scheme achieves the best risk and return trade-off, letting the Barbell Strategy capture steady returns while restraining downside risk. For risk-tolerant investors, the 15-day strategy is a feasible alternative. Despite slightly higher risk, it delivers enhanced return potential, as reflected in its concentrated high-return distribution.

## 5. Conclusions

This research develops an integrated ETF volatility forecasting framework for the Chinese stock market that couples multi-dimensional entropy indicators with machine learning models and links the predicted Yang–Zhang Volatility to a dynamically rebalanced Barbell Strategy. Based on the experiment from 2015 to 2025 across three market capitalization segments, the empirical results confirm that entropy features consistently improve forecasting performance, especially for Small-Cap ETFs, which behave as the most active and high-entropy systems and achieve the highest R Squared despite larger inherent prediction errors. Forecasting accuracy also improves with the prediction window, with the best average R Squared obtained under the 20-day window, where Shannon Entropy performs most prominently.

A central finding is the divergence between forecasting and trading efficacy. Although the Extra Trees model delivers the highest forecasting accuracy, Random Forest dominates the trading implementation, with the highest return, the lowest Maximum Drawdown, and the best Calmar Ratio. This highlights a fundamental discrepancy between forecasting optimization objectives and trading decision requirements: machine learning forecasting prioritizes the numerical accuracy of point estimates, whereas profitable trading depends more on reliable directional signals and the reasonable identification of relative volatility fluctuations. Furthermore, the 20-day rebalancing frequency, rather than the return-maximizing 15-day window, achieves the better risk–return trade-off. These results demonstrate that quantitative strategy construction requires joint trade-offs among predictive accuracy, transaction costs, and signal timeliness; overemphasizing any single objective, such as pure forecasting precision, leads to suboptimal trading performance. These findings also offer practical implications for market participants. For quantitative fund managers, the framework provides an implementable volatility-timing overlay. For asset allocators, the dynamic Barbell controls drawdowns while retaining upside. For individual investors, the strategy remains feasible at ordinary account scale since it relies solely on ETFs.

Despite these results, several limitations should be noted. First, the feature set is built on historical price, volume, and technical indicators and does not incorporate multi-source external information such as macroeconomic variables, corporate fundamentals, and social media sentiment; integrating such data may help models to capture market shocks and structural regime shifts, improving forecasting robustness. Second, we rely on traditional machine learning methods; future work can explore advanced time series deep learning models such as Transformer, TimeNet, and TCN [62] to capture the complex cross-asset interdependencies in ETF volatility dynamics. Third, the dynamic Barbell Strategy is driven primarily by volatility signals. Subsequent studies could refine asset allocation by incorporating momentum trends, sector rotation, and valuation levels to build more adaptive, intelligent trading systems. Fourth, the framework does not directly address multi-period non-overlapping volatility forecasting or the prediction of volatility changes, both of which are equally valid research perspectives; extending the entropy-based framework to multi-horizon forecasting and to volatility-regime or innovation prediction would be promising. Fifth, the current design aligns the volatility estimation window, entropy feature window, and rebalancing frequency to ensure internal consistency, so we do not separately identify each component’s marginal contribution. Sixth, the use of relatively short rolling windows introduces finite sample variability in the entropy estimates, particularly for Dispersion Entropy, where the number of observed patterns is small relative to the full state space; a comprehensive finite-sample analysis of entropy estimators and their impact on volatility forecasting would be valuable. In addition, the cross-sectional design does not lend itself to formal statistical inference because the configurations are not independent replicates. A more rigorous analysis would require daily-level forecast errors and returns, enabling methods such as Diebold–Mariano tests and block bootstrap. Finally, our empirical tests focus only on Chinese ETFs, which limits generalizability. Nonetheless, our framework can be applied to any liquid-equity market. Future work will validate this framework on American, European and other emerging markets.

## Figures and Tables

**Table 1:** The significance of entropy variables.

Stock	Variable	R Squared	MAE	MSE
Large-Cap	Baseline	0.140	2.09 × $10^{-4}$	2.73 × $10^{-7}$
	With Entropy	0.147	2.10 × $10^{-4}$	2.71 × $10^{-7}$
Mid-Cap	Baseline	0.158	2.80 × $10^{-4}$	4.57 × $10^{-7}$
	With Entropy	0.164	2.83 × $10^{-4}$	4.54 × $10^{-7}$
Small-Cap	Baseline	0.154	3.25 × $10^{-4}$	5.61 × $10^{-7}$
	With Entropy	0.162	3.28 × $10^{-4}$	5.56 × $10^{-7}$

**Table 2:** Performance for different ETFs.

Stock	R Squared	MAE	MSE
Large-Cap (Baseline)	0.75 ± 0.06	(10.1 ± 1.8) × $10^{-4}$	(5.2 ± 1.5) × $10^{-6}$
Mid-Cap (Baseline)	0.79 ± 0.05	(11.6 ± 2.0) × $10^{-4}$	(6.0 ± 1.7) × $10^{-6}$
Small-Cap (Baseline)	0.78 ± 0.06	(13.4 ± 2.3) × $10^{-4}$	(7.6 ± 2.5) × $10^{-6}$
Large-Cap	0.82 ± 0.04	(7.8 ± 1.3) × $10^{-4}$	(3.7 ± 1.0) × $10^{-6}$
Mid-Cap	0.86 ± 0.04	(8.6 ± 1.8) × $10^{-4}$	(4.2 ± 1.3) × $10^{-6}$
Small-Cap	0.86 ± 0.03	(9.6 ± 1.9) × $10^{-4}$	(4.8 ± 1.4) × $10^{-6}$

**Table 3:** Performance for different models.

Model	R Squared	MAE	MSE
AdaBoost	0.85 ± 0.03	(9.8 ± 1.2) × $10^{-4}$	(4.3 ± 0.9) × $10^{-6}$
Decision Tree	0.86 ± 0.03	(8.6 ± 1.5) × $10^{-4}$	(4.1 ± 1.2) × $10^{-6}$
Extra Trees	0.87 ± 0.02	(7.0 ± 1.1) × $10^{-4}$	(3.5 ± 0.9) × $10^{-6}$
Gradient Boosting	0.86 ± 0.03	(8.4 ± 1.4) × $10^{-4}$	(4.0 ± 0.9) × $10^{-6}$
LightGBM	0.85 ± 0.05	(8.4 ± 1.7) × $10^{-4}$	(4.1 ± 1.4) × $10^{-6}$
Random Forest	0.81 ± 0.04	(10.3 ± 2.1) × $10^{-4}$	(5.4 ± 1.8) × $10^{-6}$
XGBoost	0.84 ± 0.03	(8.0 ± 1.4) × $10^{-4}$	(4.4 ± 1.0) × $10^{-6}$

**Table 4:** Performance for different entropy indexes and windows.

Entropy	Window	R Squared	MAE	MSE
Shannon Entropy	10	0.82 ± 0.04	(10.3 ± 1.6) × $10^{-4}$	(5.5 ± 1.4) × $10^{-6}$
	15	0.85 ± 0.03	(8.4 ± 1.3) × $10^{-4}$	(4.1 ± 0.8) × $10^{-6}$
	20	0.88 ± 0.02	(7.3 ± 1.3) × $10^{-4}$	(3.1 ± 0.6) × $10^{-6}$
Fuzzy Entropy	10	0.82 ± 0.04	(10.1 ± 1.5) × $10^{-4}$	(5.4 ± 1.3) × $10^{-6}$
	15	0.85 ± 0.03	(8.4 ± 1.4) × $10^{-4}$	(4.1 ± 0.8) × $10^{-6}$
	20	0.87 ± 0.03	(7.3 ± 1.3) × $10^{-4}$	(3.2 ± 0.6) × $10^{-6}$
Permutation Entropy	10	0.82 ± 0.04	(10.3 ± 1.7) × $10^{-4}$	(5.4 ± 1.3) × $10^{-6}$
	15	0.85 ± 0.03	(8.4 ± 1.2) × $10^{-4}$	(4.1 ± 0.7) × $10^{-6}$
	20	0.87 ± 0.03	(7.3 ± 1.3) × $10^{-4}$	(3.2 ± 0.6) × $10^{-6}$
Dispersion Entropy	10	0.82 ± 0.04	(10.2 ± 1.5) × $10^{-4}$	(5.4 ± 1.2) × $10^{-6}$
	15	0.84 ± 0.04	(8.5 ± 1.4) × $10^{-4}$	(4.2 ± 0.8) × $10^{-6}$
	20	0.87 ± 0.03	(7.3 ± 1.3) × $10^{-4}$	(3.2 ± 0.6) × $10^{-6}$

**Table 5:** Performance of the benchmark strategy.

Stock	Return (%)	Annualized Return (%)	Maximum Drawdown (%)	Calmar Ratio
Portfolio (Large-Cap)	46.5	5.8	29.6	0.20
Portfolio (Mid-Cap)	61.3	7.4	26.9	0.27
Portfolio (Small-Cap)	56.3	6.9	30.5	0.23
Large-Cap	56.0	6.8	45.6	0.15
Mid-Cap	80.7	9.2	41.8	0.22
Small-Cap	72.3	8.4	46.7	0.18

**Table 6:** Trading performance for different ETFs and frequencies.

Stock	Frequency	Return (%)	Annualized Return (%)	Maximum Drawdown (%)	Calmar Ratio
Large-Cap	10	26.4 ± 2.2	3.5 ± 0.3	21.9 ± 0.8	0.16 ± 0.02
	15	42.6 ± 1.0	5.4 ± 0.1	25.7 ± 0.4	0.21 ± 0.01
	20	28.7 ± 1.8	3.8 ± 0.2	18.9 ± 0.3	0.20 ± 0.01
Mid-Cap	10	23.5 ± 2.9	3.2 ± 0.4	26.0 ± 0.9	0.12 ± 0.02
	15	49.8 ± 1.7	6.2 ± 0.2	24.1 ± 0.4	0.26 ± 0.01
	20	43.8 ± 2.3	5.5 ± 0.3	17.2 ± 0.3	0.32 ± 0.01
Small-Cap	10	27.9 ± 2.4	3.7 ± 0.3	28.7 ± 1.6	0.13 ± 0.02
	15	49.1 ± 0.8	6.1 ± 0.1	24.8 ± 0.5	0.25 ± 0.01
	20	45.8 ± 2.1	5.8 ± 0.2	19.5 ± 0.3	0.30 ± 0.01

**Table 7:** Trading performance for different models.

Model	Return (%)	Annualized Return (%)	Maximum Drawdown (%)	Calmar Ratio
AdaBoost	37.0 ± 10.6	4.7 ± 1.2	23.1 ± 3.9	0.21 ± 0.07
Decision Tree	37.5 ± 11.2	4.8 ± 1.3	23.5 ± 4.0	0.21 ± 0.07
Extra Trees	39.1 ± 9.5	5.0 ± 1.1	23.1 ± 3.7	0.22 ± 0.07
Gradient Boosting	36.3 ± 10.9	4.7 ± 1.2	23.0 ± 3.7	0.21 ± 0.07
LightGBM	37.2 ± 10.0	4.8 ± 1.1	23.0 ± 3.6	0.21 ± 0.06
Random Forest	39.4 ± 8.8	5.0 ± 1.0	22.0 ± 2.9	0.23 ± 0.06
XGBoost	36.1 ± 10.2	4.6 ± 1.2	23.1 ± 3.8	0.21 ± 0.07

**Table 8:** Trading performance for different entropy indexes and frequencies.

Entropy	Frequency	Return (%)	Annualized Return (%)	Maximum Drawdown (%)	Calmar Ratio
Shannon Entropy	10	26.0 ± 3.3	3.5 ± 0.4	25.5 ± 3.1	0.14 ± 0.03
	15	47.0 ± 3.5	5.9 ± 0.4	24.8 ± 0.9	0.24 ± 0.02
	20	39.3 ± 7.9	5.0 ± 0.9	18.5 ± 1.0	0.27 ± 0.05
Fuzzy Entropy	10	25.7 ± 3.1	3.5 ± 0.4	25.6 ± 3.1	0.14 ± 0.03
	15	47.1 ± 3.4	5.9 ± 0.4	24.9 ± 0.8	0.24 ± 0.02
	20	39.5 ± 7.9	5.0 ± 0.9	18.5 ± 1.0	0.27 ± 0.05
Permutation Entropy	10	25.9 ± 3.2	3.5 ± 0.4	25.4 ± 2.9	0.14 ± 0.02
	15	47.2 ± 3.1	5.9 ± 0.3	24.9 ± 0.7	0.24 ± 0.02
	20	39.5 ± 8.1	5.0 ± 0.9	18.5 ± 1.0	0.27 ± 0.05
Dispersion Entropy	10	26.1 ± 2.7	3.5 ± 0.3	25.6 ± 3.1	0.14 ± 0.02
	15	47.4 ± 3.8	5.9 ± 0.4	24.8 ± 0.8	0.24 ± 0.02
	20	39.3 ± 7.8	5.0 ± 0.9	18.6 ± 1.0	0.27 ± 0.05

## Data Availability

The data presented in this research are available on request from the corresponding author.

## Abbreviations

The following abbreviations are used in this manuscript:

ETF	Exchange-Traded Fund
AR	Autoregressive
MA	Moving Average
ARIMA	Autoregressive Integrated Moving Average
GARCH	Generalized Autoregressive Conditional Heteroskedasticity
NN	Neural Network
GRU	Gated Recurrent Unit
LSTM	Long Short-Term Memory
TCN	Temporal Convolutional Network
CNN	Convolutional Neural Network
HAR	Heterogeneous Autoregressive
VMD	Variational Mode Decomposition
MACD	Moving Average Convergence Divergence
EMA	Exponential Moving Average
ATR	Average True Range
HV	Historical Volatility
RMSE	Root Mean Squared Error
MAE	Mean Absolute Error
MSE	Mean Squared Error
ANOVA	One-Way Analysis of Variance
